# PBHoney: identifying genomic variants via long-read discordance and interrupted mapping

**DOI:** 10.1186/1471-2105-15-180

**Published:** 2014-06-10

**Authors:** Adam C English, William J Salerno, Jeffrey G Reid

**Affiliations:** 1 Human Genome Sequencing Center at Baylor College of Medicine, One Baylor Plaza, Houston 77030, Texas, USA

**Keywords:** Structural variation, Sequencing, PacificBiosciences

## Abstract

**Background:**

As resequencing projects become more prevalent across a larger number of species, accurate variant identification will further elucidate the nature of genetic diversity and become increasingly relevant in genomic studies. However, the identification of larger genomic variants via DNA sequencing is limited by both the incomplete information provided by sequencing reads and the nature of the genome itself. Long-read sequencing technologies provide high-resolution access to structural variants often inaccessible to shorter reads.

**Results:**

We present PBHoney, software that considers both intra-read discordance and soft-clipped tails of long reads (>10,000 bp) to identify structural variants. As a proof of concept, we identify four structural variants and two genomic features in a strain of *Escherichia coli* with PBHoney and validate them via *de novo* assembly. PBHoney is available for download at http://sourceforge.net/projects/pb-jelly/.

**Conclusions:**

Implementing two variant-identification approaches that exploit the high mappability of long reads, PBHoney is demonstrated as being effective at detecting larger structural variants using whole-genome Pacific Biosciences RS II Continuous Long Reads. Furthermore, PBHoney is able to discover two genomic features: the existence of Rac-Phage in isolate; evidence of *E. coli*’s circular genome.

## Background

Structural variation results from numerous biological processes and has been implicated in a variety of diseases and phenotypes (see for review [1-5]). As resequencing projects become more prevalent across a larger number of species, accurate variant identification will further elucidate the nature of genetic diversity and become increasingly relevant in genomic studies. However, the identification of structural variants via DNA sequencing is limited by both the incomplete information provided by sequencing reads and the nature of the genome itself.

Next-generation sequencing (NGS) technologies generate reads ranging from dozens to hundreds of base pairs (bp) in length and with relatively low per-base error rates. Moreover, many NGS technologies generate sets of coordinated reads whose genomic separation is known a priori (e.g., paired-end and mate-pair reads). When reads generated from a sample sequence are aligned to a reference genome sequence, variation between the sample and reference genomes manifests itself as imperfect mapping. NGS genomic variation detection methods take advantage of different types of imperfect mappings to detect different variant types. Variants smaller than the read length (traditionally single-nucleotide variants and indels) are identified via discordance (i.e., mismatches and gaps) between a sample read and the reference sequence [6]. Longer, structural variants include copy-number variants, inversions, and translocations. Depth-of-coverage methods infer copy-number variants from regions of non-uniform mapping coverage [7,8]. Split-read and paired-end methods both use reads or pairs of reads that map non-contiguously to characterize genomic rearrangements larger than the read itself [9,10].

Mapping errors caused by genomic variation are difficult to distinguish from those introduced by sequencing errors and repetitive genomic sequence. Although NGS sequencing error rates are relatively low and their effects can often be mitigated with increased genomic coverage, repetitive sequence still creates mapping ambiguity. Repetitive regions of the genome also exacerbate the search for genomic variation because many variants occur in these regions [11]. Moreover, NGS methods do not always completely characterize large structural variants, often failing to provide full base-pair resolution of the entire region of interest. Finally, the efficacy of non-contiguous NGS methods can vary for different types of genomic variants depending on the sample data characteristics, such as insert-size distribution and coverage [12].

We can mitigate these limitations by taking advantage of continuous long reads generated by the Pacific Biosciences (PacBio) RS Sequencer. Each such read is a fully resolved sequence up to 30,000 bp. Despite a relatively high per-base error rate (∼15%), PacBio reads lack systematic biases and can be mapped with high accuracy [13]. In the present work, we describe two methods of identifying larger genomic variants via PacBio sequencing: interrupted mapping (PBHoney-Tails) and intra-read discordance (PBHoney-Spots). While these methods parallel NGS methods conceptually, the longer PacBio reads can be more accurately mapped and can span larger genomic variants. And, unlike previously published approaches to finding structural variants with ’long’ reads [14,15], PBHoney is designed to handle continuous long reads with lower base-accuracy.

As proof of concept, we applied PBHoney to PacBio reads generated from *Escherichia coli* and identified four structural and two genomic features, each of which was confirmed via *de novo* assembly.

## Implementation

### Interrupted long-read mapping

**Figure 1:**
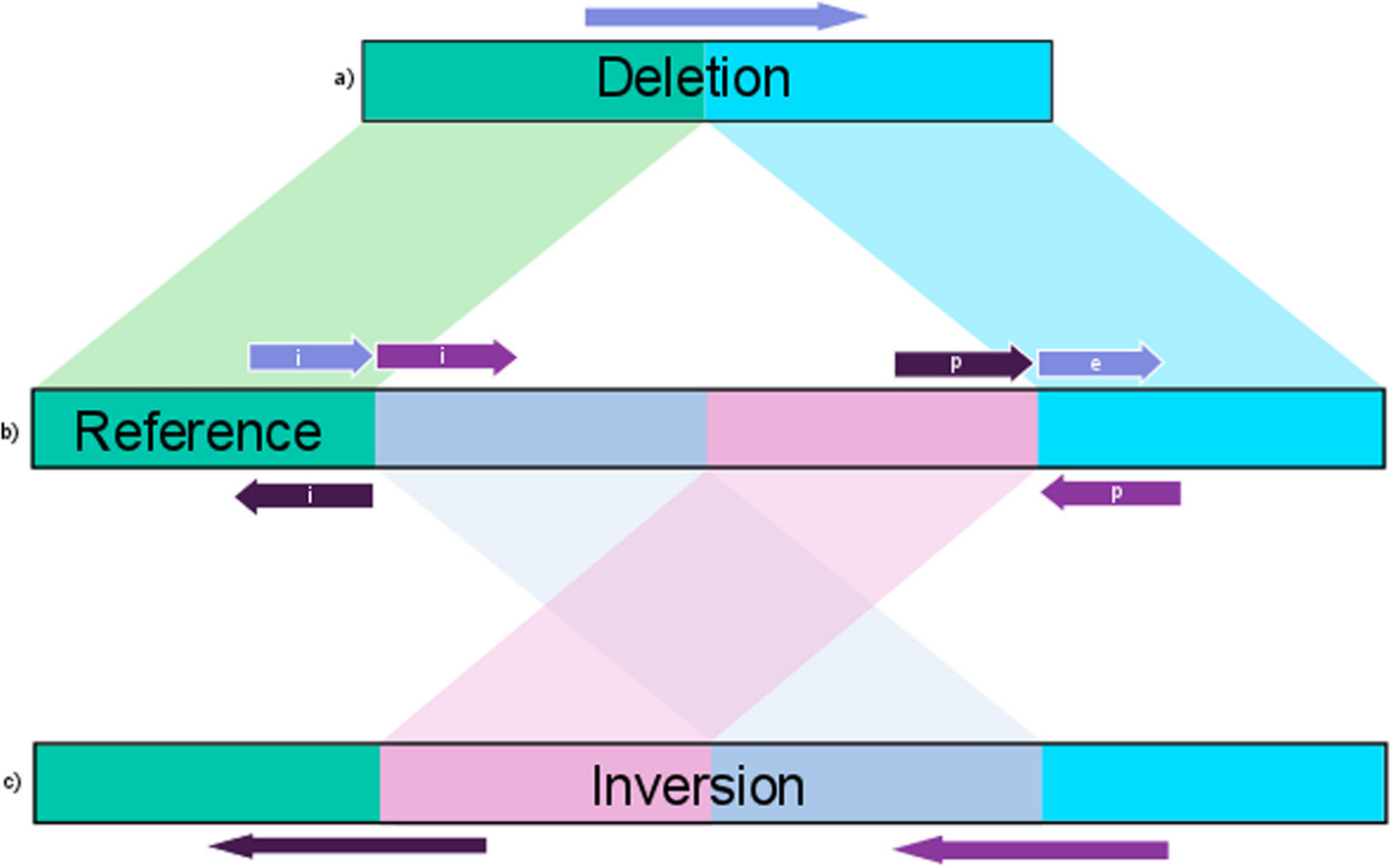
**Tail Schematic.** Schematic of possible tails created by reads representing a deletion **(a)** and an inversion **(c)** allele and the structural variants they represent when mapped to the reference **(b)**. Rectangles represent double-stranded genomic sequence. Arrows above and below a rectangle represent reads mapping to the direct and complement strands, respectively. In these examples, all initial alignments align at the 5’ breakpoint of the reference. The read spanning a deletion event creates an epilog that maps to the 3’ breakpoint on the same strand as its corresponding initial alignment. The reads spanning the inversion event breakpoints create prologs that map on the opposite strands of their corresponding initial alignment. While all three piece-alignments would cluster if we considered only their location, their orientations support two separate events in the reference region.

PacBio RS filtered subreads are first mapped to a reference genome with BLASR [13], an alignment tool optimized for reads thousands of base pairs long with higher error rates. The BLASR output is a SAM alignment [16] that contains each read’s single best alignment. Any such best alignment does not necessarily map each read position to the reference: the mapped read can be truncated prior to the 5’ and 3’ ends, creating an interrupted mapping represented by soft-clipped (i.e., unmapped) tails. In the present work, all tails longer than 200 bp are extracted from the SAM alignment and remapped to the reference genome with BLASR, which reports each tail’s best alignment. Thus, any mapped read comprises an initial alignment and up to two mapped tails, a 5’ prolog and a 3’ epilog, which when taken together compose a piece-alignment. These piece-alignments are placed in a new SAM file that contains tags for each alignment describing the locations and orientations of other members of the same piece-alignment. The piece-alignments of most filtered subreads comprise only an initial alignment, while only a small subset of these reads produce both prologs and epilogs.We next cluster piece-alignments with similarly mapped tails based on location and orientation. Figure 1 describes two sets of possible tail locations and orientations. Here, we only consider up to two components of piece-alignments (i.e., a prolog and initial or an initial and an epilog) because piece-alignments with more than two components due to structural variation and not low-quality sequence are rare. Should a read produce both a prolog and epilog, the alignment with the higher mapping quality is chosen.First, two piece-alignments are candidates for clustering if the corresponding component alignments have locations that support breakpoints at similar positions by beginning and ending at a distance less than a user-defined buffer length. Buffer length is set at 200bp for this work and by default within the software. Second, to form a cluster, the piece-alignments must share the same internal orientation of component alignments. By only clustering events that satisfy both conditions, we can distinguish multiple variants that may share similar breakpoints, as is the case in the Figure 1 example.

Each final cluster can contain any number of participating piece-alignments (e.g., a single read with a mapped tail is considered a cluster). Using mapping orientations and location, we then annotate each cluster as a deletion, insertion, inversion, or translocation and predict breakpoints as the average interrupted position of each read. In this study we only report clusters with a minimum of three piece-alignments and a minimum average Phred-scale mapping quality value of 100. These minima exclude piece-alignments that are possibly the result of chimeras in the sample preparation and short, non-confidently mapped reads.

### Intra-read discordance

PacBio RS reads have an experimentally determined 15% per-base error rate but lack systematic errors such as GC bias [17,18]. Because the errors are stochastic, we can identify discordant “spots” within the reference where the error rate is higher than expected. Using the final SAM file (which includes previously unmapped tails), we count the number of errors at every position in the reference. At any such position, each aligned subread can agree with the reference or produce a mismatch, deletion, or insertion. Each of these error ’channels’ (mismatches, deletions, and insertions) and coverage is calculated and stored in a 4×*G* integer array (*A*), where *G* is length of the reference. To identify regions of discordance we convolve the array with several kernels. First, we obtain the error rate at each position by dividing the error channels by the coverage at each position:

$$E_{\text{ji}}=A_{\text{ji}}/C_{i},$$

where *A*_*j* *i*_ is the value of the *j*th channel at position *i* in the reference and *C*_*i*_ is the coverage at that position. Next, we apply a smoothing kernel that replaces each value in the array with the mean channel value over a window of length 2*B*+1 centered at the associated genomic position *i*. Formally,

$$M_{\text{ji}}=\frac{1}{2B+1}\overset{i+B}{\underset{k=i-B}{\sum }}E_{\text{jk}}.$$

We then obtain the standard deviation and mean for each channel across the whole chromosome. Finally, we calculate changes in discordance on a per-window basis by applying a slope kernel:

$$S_{\text{ji}}=\frac{1}{B}\left(\overset{i-1}{\underset{k=i-B}{\sum }}M_{\text{jk}}-\overset{i+B}{\underset{k=i+1}{\sum }}M_{\text{jk}}\right).$$

**Figure 2:**
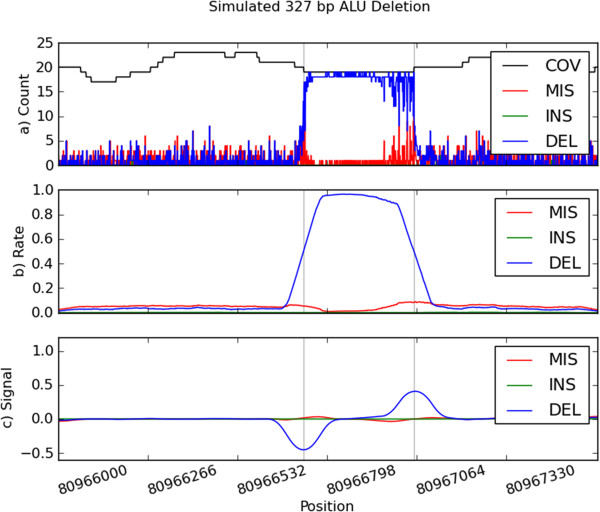
**Simulated ALU Deletion.** Plot **(a)** depicts the raw channels for the 327 bp ALU Deletion. Raw channels include coverage (COV), mismatches (MIS), insertions (INS), and deletions (DEL). Plot **(b)** are the channels after smoothing, and plot **(c)** is the final signal after applying the slope kernel. The gray lines represent the start and end points of the deletion.

Each array value now measures the extent to which the channel values before each position differ from the channel values after. Figure 2 illustrates the signal processing for a simulated ALU deletion [11].

Using the above channels, we identify possible structural variants by extracting regions that contain increases in discordance (negative *S*_*j* *i*_ values) followed by decreases in discordance (positive *S*_*j* *i*_ values), corresponding to the starts and ends of genomic variants, respectively. To do so, we set discordance thresholds to N times each channel’s standard deviation, where N is a user-defined parameter with an empirically determined default of 5. For each channel, we then extract ’peaks’ that sit above these thresholds. The widths of these peaks determine the outer and inner boundaries for the variant breakpoints. Furthermore, we predict an exact breakpoint as the point of maximum discordance in the outer and inner boundaries. Thus, a possible genomic variant is reported as two sets of genomic coordinates, (*start*_*i* *n*_,*start*,*start*_*o* *u* *t*_) and (*end*_*i* *n*_,*end*,*end*_*o* *u* *t*_) and a type determined by the channel (insertion, deletion, mismatch). These boundary coordinates allow us to account for the low base-error and realignment issues (such as repeats) that occur near most structural variants.

## Results

We generated DNA from *E. coli* K12 strain MG1655 and created a 17 Kbp mean DNA insert-size using a Blue-Pippin preparation protocol (as recommended by Pacific Biosciences). The filtered subreads produced were on average 6.1 Kbp long (8.7 Kbp N50) and had a mean accuracy of 86.4% when mapped to the *E. coli* reference genome (GenBank accession U00096.2). A total of 95,778 PacBio RS filtered subreads were generated with an average length of 6.1 Kbp and an N50 length of 8.75 Kbp, providing an expected 126X average coverage of the 4.6 Mbp *E. coli* genome.

PacBio reads are capable of creating high quality assemblies [19]. Therefore, before detecting variants, we assembled the PacBio reads to create a sample reference genome using the same nonhybrid HGAP assembly techniques to independently discover variants. The sample reference genome comprised five contigs that uniquely covered 84.8% of the *E. coli* reference genome with an N50 of 1.5 Mbp. We then used MUMmer [20] to identify all variants greater than 100 bp between the newly assembled sample reference and the standard *E. coli* reference. This analysis identified a transposon deletion, a tandem duplication, and a tandem deletion.

After mapping the reads to the *E. coli* genome and processing the alignment through PBHoney, we discovered evidence of *E. coli*’s circular genome, four structural variants, and evidence of Rac phage in the *E. coli* culture.

### Transposon deletion

PBHoney identified a deletion with breakpoints at coordinates 1,976,520 and 1,977,300. With a length of less than 1,000 bp, this deletion is small enough for some PacBio reads to accurately map to the unvarying flanking sequence of the deletion in the reference, much in the same way that a mapped NGS read can span a small indel. While some PacBio reads are not long enough to span the deleted sequence, many of these reads’ tails are long enough to map to the opposite side of the deletion.

### Tandem duplication and deletion

We identifed an insertion of approximately 180 bp between the coordinates 1,096,766 and 1,096,817, and using the PacBio ALLORA assembly engine (Pacific Biosciences Menlo Park, CA), we resolved the full insertion sequence by assembling the reads that mapped to that region.

To confirm the tandem nature of this insertion, we used Tandem Repeats Finder (TRF) [21] to identify 3.4 copies of a 181 bp repeat present in that region of the reference genome. When applied TRF to the assembly of sample reads and 4.4 copies were reported.

Similarly, we identified a 113 bp deletion in the reference between the coordinates 4,294,274 and 4,294,369. Applying the same methods, we found 5.3 copies of the repeat in the reference genome and 4.3 copies in the sample assembly. By remapping this assembly to the reference genome, we found the deletion to sit between the coordinates 4,294,294 and 4,294,405.

### P-Element inversion

The e14 prophage of the *E. coli* genome contains a 1,828 bp invertible P-element [22]. While this variant was too long to be spanned by mapped reads, we identified a subset of reads in the region that map in a manner suggesting an inversion between the coordinates 1,207,027 and 1,208,827.

These coordinates differ from the EcoGene (http://www.ecogene.org) annotated location of the inversion (1,207,013 and 1,208,841). This difference is attributable to the inverted repeats that flank the P-element and create alignment ambiguity (i.e., aligning query bases to one copy of the repeat instead of the other). This ambiguity is overcome by shifting bases to a single copy of the repeat, which recreates the exact annotated breakpoints.

Because the P-element inversion only occurs in a subset (28 reads in 133x coverage) of the *E. coli* organisms in a given culture, *de novo* assembly does not expose the event. However, our results allow us to easily identify the reads that do support the variant. By performing an assembly using the subset of reads we recovered the full inverted sequence.

### Rac prophage

In addition to genomic variants inside the *E. coli* genome, we found 8 reads that had interrupted mapping at the boundaries of *E. coli*’s Rac prophage genomic feature. PBHoney annotated this event as a reference genome insertion. However, a more complete annotation is that these reads are the result of the defective bacteriophage’s replication and its genome existing in isolate in our sample. When we assembled the reads that supported this event, we recovered the 25,556 bp circular genome sequence of the phage.

### Performance

To assess how well PBHoney performs with lower coverage, we ran PBHoney on alignments down-sampled to 10X, and 20X coverage fifty times per coverage with default parameters. We repeated this experiment four times while increasing and decreasing independently spots’ minimum coverage parameter and mimimum standard-deviation threshold parameter. We then assessed PBHoney’s ability to detect structural variants at each run by comparing the detected variants to the four known variants and evidence of the circular genome (Table 1). It should be noted that with default parameters, 21 and 5 false negatives at 10 × and 20 × coverage respectively can be attributed to the missing P-element inversion. These false negatives are because the P-element inversion only occurs in a minority of *E. coli* organisms (∼25%) and therefore isn’t guaranteed to be represented in the down-sampled coverage. If we exclude the P-element inversion from our truth set, 10X coverage’s sensitivity increases to 90.5% and 20x coverage to 94.5%.

**Table 1:** Performance over 50 down-sampling experiments at 10X, and 20X coverage

**Coverage**	**10x**					**20x**				
**Params**	**"c5 e5"**	**"c7 e5"**	**"c5 e7"**	**"c3 e5**"	**"c5 e3"**	**"c5 e5"**	**"c7 e5"**	**"c5 e7"**	**"c3 e5"**	**"c5 e3"**
TP	210	182	187	217	210	234	239	228	237	236
FP	25	0	7	55	302	1	0	1	3	46
FN	40	68	63	40	38	16	11	22	13	14
Sensitivity	84.00%	72.80%	74.80%	86.80%	84.00%	93.60%	95.60%	91.20%	94.80%	94.40%
PPV	89.36%	100.00%	96.39%	79.78%	41.02%	99.57%	100.00%	99.56%	98.75%	83.69%

True Positive (TP) False Positive (FP) False Negative (FN) counts, Sensitivity, and Positive Predictive Value (PPV). PPV is the probability any given variant call is true (TP/(TP+FP)). Parameters changed are Coverage (c) and Standard-deviation Threshold (e) for spots signal processing.

To benchmark the computational performance of PBHoney, we re-ran our *E.coli* dataset three times. On average, PBHoney spots was able to process the 93k reads in 27 minutes with a peak memory usage of 232mb and an average usage of 119mb. PBHoney tails processed reads on average in 28 seconds with a peak memory usage of 177mb and average usage of 90mb. In order to estimate a maximum resource usage of PBHoney, we tested 10x coverage (600k reads) simulated using blasr’s alchemy program over Human chromosome 1 through spots processing and found a peak memory usage of 10.4gb (2gb average) over 3.5 hours of processing time.

## Discussion

If DNA sequencing technologies could produce a single read of chromosome or genome length, variant identification would be a matter of comparing two similar strings. Such methodologies are already being applied in comparative genomics structural studies [23] and *de novo* assembly methods of structural variation discovery [24]. However, given the computational challenges of whole genome *de novo* assembly, variant identification is limited by our ability to accurately map sample reads to the reference genome.

Many factors contribute to whether a read will span a variant region or create an interrupted mapping, including the length and quality of the read, the register of the read relative to the variant region, the mappability and size of the variant region, and the nature of the alignment algorithm. The length and per-base error rates of PacBio reads allow structural variants to ’hide’ inside of the noise of the stochastic errors. For example, a 500 bp deletion in the sample relative to the reference can be spanned by a 10 Kbp read because 500 additional ’errors’ in the mapping of a 10 Kbp with 1500 expected errors can be insufficient to interrupt the mapping. Such hidden variants create intra-read discordance and are revealed by PBHoney-Spots. If the variant does create an interrupted mapping, PBHoney-Tails leverages this information to characterize the variant. By incorporating these two distinct methods, PBHoney is insensitive to how a read maps to the reference, much as are NGS methods that use both paired-end and split-read information. PBHoney also limits itself to categorizing these variants in the context of our *in silico* abstraction of a genomic variant as a local string comparison.

We have also presented an exploration of the parameter space by repeating out titration experiments with changes in the minimum coverage and minimum standard-deviation threshold parameters. Other parameters available for the user include the minimum size of a tail to be considered for remapping, the minimum number of tailed reads needed to support a call, the minimum number of unique tailed reads needed to support (i.e. from different zero-mode-waveguides - this helps remove false-positives that are the result of chimeras), the minimum mapping quality of a read and it’s tail to be included in a variant call, and the minimum size of a structural variant. Since the most time consuming step in spots processing is counting the errors at any particular base (2.8 of 3.5 hours in the Human chromosome 1 simulation test), an hdf5 file is stored containing the arrays necessary to reprocess with different parameters should the user wish to tweak his or her spots results quickly.

In the present work, PBHoney reports the breakpoint location and a mapping-based classification of each variant as one of insertion, deletion, mismatch, inversion, and translocation. These results are sufficient to identify reads for reassembly, from which the full sequence of the event and exact breakpoints can be recovered. More complex and biologically informed classification thus becomes an independent and subsequent step to mapping-based annotation. Samples with more biologically complex variants still manifest themselves through the methods presented here, and when variants are considered in a global context, the complex variation can be reconstructed. Future versions of PBHoney will automate the assembly process and include more sophisticated variant classification that uses existing variant-specific tools such as Tandem Repeats Finder and novel haplotype reconstruction software to further elucidate the specific variant types that occur.

For this work’s proof of concept, we processed the haploid *E. coli* genome and therefore did not include genotyping information in our calls. However, estimates of genotype can currently be established by looking at the coverage of reads that support an alternate allele versus supporting the reference. Future work will include automating this procedure.

## Conclusions

Genomic variation detection faces many challenges when creating a completely characterized genome with identified large and complex variants. This work describes PBHoney, which leverages the high mappability of long reads to identify structural variants in a manner similar to the split-read and paired-end methods applied to shorter reads. The first continuous long-read specific structural variant software, PBHoney should prove valuable to resequencing efforts, particularly with regions inaccessible to short-read read mapping, specifically genomic regions subject to repetitive elements that are known to enrich for large variation events.

## Availability and requirements

**Project name:** PBHoney**Project home page: **http://sourceforge.net/projects/pb-jelly**Operating system(s):** Platform independent**Programming language:** Python**Other requirements:** Python 2.7, samtools 0.1.17, blasr 1.3.1, h5py 2.0.1, pysam 0.7.4, numpy 1.6**License:** FreeBSD.

## Competing interests

The authors declare that they have no competing interests.

## Authors’ contributions

ACE designed and performed the experiments, developed the code, and contributed to writing the manuscript. WJS contributed to experimental design and wrote the manuscript. JGR contributed to writing the manuscript and designing the experiments. All authors read and approved the final manuscript.
